# Human-to-Human Transmission of Andes Virus Modeled in Syrian Hamsters

**DOI:** 10.3201/eid2910.230544

**Published:** 2023-10

**Authors:** Silke A. Riesle-Sbarbaro, Norman Kirchoff, Katharina Hansen-Kant, Alice Stern, Andreas Kurth, Joseph B. Prescott

**Affiliations:** Robert Koch Institute, Berlin, Germany

**Keywords:** Andes virus, Hantavirus, Hantavirus cardiopulmonary syndrome, Syrian hamster, viruses, Germany

## Abstract

Several occurrences of human-to-human transmission of Andes virus, an etiological agent of hantavirus cardiopulmonary syndrome, are documented. Syrian hamsters consistently model human hantavirus cardiopulmonary syndrome, yet neither transmission nor shedding has been investigated. We demonstrate horizontal virus transmission and show that Andes virus is shed efficiently from both inoculated and contact-infected hamsters.

Hantavirus cardiopulmonary syndrome (HCPS) is a sporadic, lethal (>40% fatality rates) zoonotic disease, which in South America is primarily caused by Andes virus (ANDV) (*1*). HCPS is prevalent where the natural rodent reservoir, the long-tailed colilargo (*Oligoryzomys longicaudatus*), is present. Zoonotic transmission was thought to occur exclusively through exposure to aerosolized infectious particles from excreta or secreta of infected reservoirs (*2*). However, unique to ANDV among hantaviruses, person-to-person transmission events have been described, highlighting the potential importance of onward transmission for outbreaks (*3*–*5*). 

Syrian hamsters (*Mesocricetus auratus*) infected with ANDV uniquely mimic many aspects of humans with ANDV-HCPS disease (*6*,*7*). This model has been crucial for understanding HCPS immunopathology and for developing potential therapeutic treatments (*8*–*11*). Although disease modeling in hamsters has been characterized extensively, studies of virus shedding and transmission are absent. In this study, we investigated whether horizontal transmission can be modeled in ANDV-infected hamsters.

## The Study

To model ANDV shedding and transmission between hamsters, we placed 6 pairs of intranasally-inoculated (200 PFU equivalences of ANDV-9717869) hamsters in clean cages 1 day postinoculation (dpi). To increase contact events, we then introduced 6 pairs of naive hamsters (i.e., contacts) to the inoculated hamsters (6 cages) (Appendix Figure 1). Infectious work was performed within the Biosafety Level 4 facility at the Robert Koch Institute (Berlin, Germany). Animal experiments were approved by Landesamt für Gesundheit und Soziales (permit no. G0142/21). Approval of animal experimentation within Biosafety Level 4 facilities at the Robert Koch Institute was granted by the Regional Office for Health and Social Affairs, Berlin.

We implanted all hamsters with temperature-logging transponders (IPTT-300 Temperature Transponder; Plexx, https://www.plexx.eu). Throughout the experiment, we observed hamsters daily to assess disease signs. Pathognomonic acute disease signs (*6*,*11*) were a criterion for euthanasia, and surviving animals were euthanized at 40 dpi. At 27 dpi, one naive animal (c1-n1) was euthanized for unrelated illness (Table 1). Under isoflurane sedation, all animals were routinely weighed and sampled (oral and rectal mucosa and, opportunistically, urine). We collected blood and tissue samples at euthanasia. ANDV RNA copies and nucleocapsid IgG were measured as reported (*10*).

**Table 1:** Study design and disease progression in study of human-to-human transmission of Andes virus modeled in Syrian hamsters*

Cage no.	Inoculated	Ab titer	Status	Naive	Ab titer	Status
1	c1-i1	≥51200	Infected	c1-n1	Excluded	
	c1-i2	3200	Disease	c1-n2	12800	Disease
2	c2-i1	200	Disease	c2-n1	3200	Disease
	c2-i2	3200	Disease	c2-n2	Negative	Infected
3	c3-i1	12800	Disease	c3-n1	Negative	Uninfected
	c3-i2	800	Disease	c3-n2	Negative	Uninfected
4	c4-i1	200	Disease	c4-n1	Negative	Uninfected
	c4-i2	3200	Disease	c4-n2	Negative	Uninfected
5	c5-i1	3200	Disease	c5-n1	Negative	Uninfected
	c5-i2	3200	Disease	c5-n2	3200	Disease
6	c6-i1	12800	Disease	c6-n1	Negative	Uninfected
	c6-i2	≥51200	Recovered	c6-n2	3200	Infected
Total infected			100%	45%		
Total HCPS			83%	27%		

*AB, antibody; HCPS, Hantavirus-induced cardiopulmonary syndrome.

**Figure 1:**
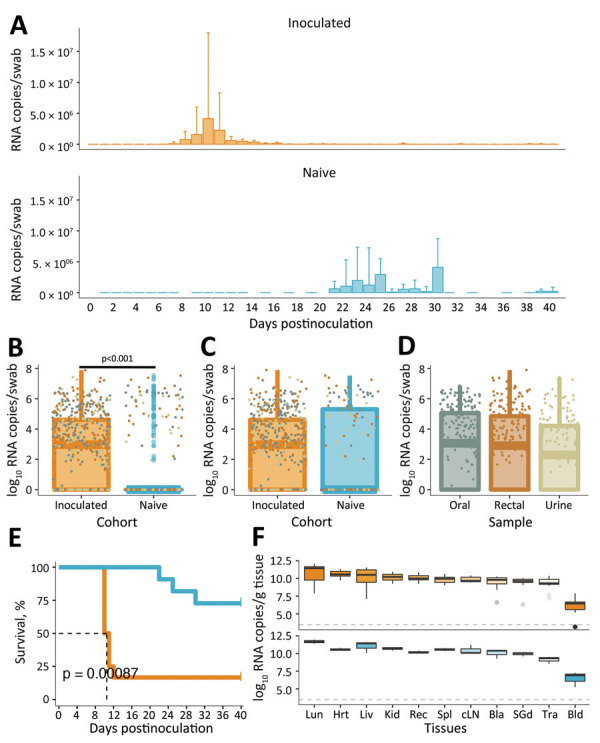
Dissemination and shedding of Andes virus (ANDV) in secretions and excretions of infected Syrian hamsters. A) ANDV RNA small segment loads in oral and rectal mucosa and urine sampled per day postinoculation throughout the experiment. B–C) Total ANDV RNA detected in all shedding routes (oral and rectal mucosa and urine), compared between cohorts (B) and between infected animals of each cohort (C) after adjustment of incubation days (AID). D) Comparison of ANDV RNA load between oral and rectal mucosa samples and urine samples from infected animals, using AID. Individual oral, rectal and urine samples are shown as points. Geometric mean RNA loads are displayed for log_10_ transformed data in panels B–D; error bars indicate SDs. E) Statistical analysis of survival between cohorts. Significance of Mantel-Cox log-rank test is shown within the plot (p<0.001). F) ANDV RNA distribution shown per gram of tissue or milliliter of blood. As reference, the horizontal grey line shows the inoculum dose. Bla, bladder; Bld, blood; cLN, cervical lymph node; Hrt, heart; Kid, kidney; Liv, liver; Lun, lung; Rec, rectum; SGd, submandibular salivary gland; Spl, spleen; Tra, trachea.

**Figure 2:**
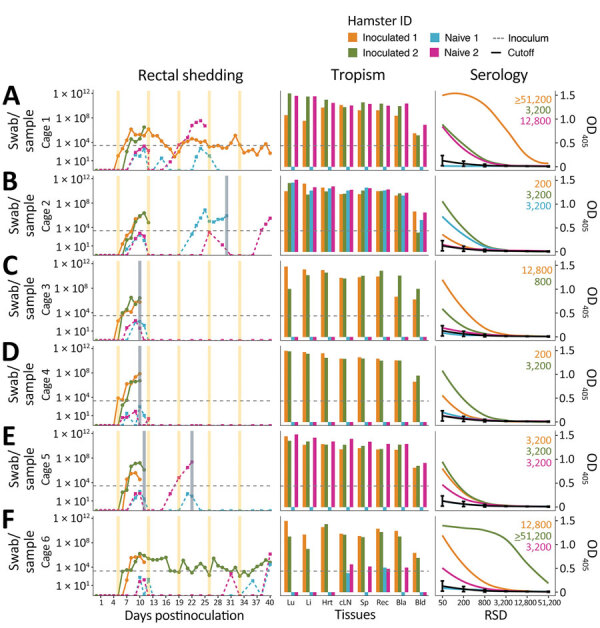
Timeline of Andes virus (ANDV) shedding and transmission between inoculated and naive Syrian hamster pairs from each cage. Panels A–F show data for cages 1–6. Left column displays shedding of ANDV RNA loads per rectal mucosa swab sample. Shedding loads of individual animals are shown as color-coded lines. Vertical shades show routine (yellow) or extra (grey) cage changes. Middle column displays tissue distribution of ANDV RNA per gram of tissue or milliliter of blood. The dashed horizontal grey line shows the inoculum dose. Right column displays results of nucleocapsid ELISA of serum collected at euthanasia. Antinucleocapsid serum titers are noted for animals that seroconverted. The assay cutoff is shown as a black curve with vertical line-ranges (mean +3 SD) of each serum dilution. To improve figure visualization, the y-axes in panels A–C were log_10_-transformed. Bla, bladder; Bld, blood; cLN, cervical lymph node; Hrt, heart; ID, identification; Li, liver; Lu, lung; OD_405_, Optical density at 405 nm; Rec, rectum; RSD, reciprocal serum dilutions; Sp, spleen.

Irrespective of inoculation route, infected hamsters typically succumb uniformly to disease (*6*); however, 16.7% of the inoculated cohort survived, potentially because of the lower intranasal infection efficacy (>8-fold inoculum dose required) than that of an intramuscular infection (Table 1). Hamster c6-i2 recovered from moderate disease, and hamster c1-i1 remained healthy. All inoculated hamsters seroconverted (Table 1) and shed abundant ANDV RNA through tested routes until being euthanized (Figure 1, panels A–D; Figure 2; Appendix Figure 2, panel A). Onset of shedding averaged 6 dpi (5 dpi orally and 6 dpi rectally and in urine). Shedding was detected as early as 1 dpi in oral mucosa and urine, and peak shedding occurred consistently 1 day before euthanasia. Horizontal ANDV transmission was evidenced in 45% of the naive cohort (5/11 contacts), but HCPS developed in only 3 hamsters before the predetermined experimental endpoint (Table 1). Survival was significantly different between cohorts (Figure 1, panel E); inoculated hamsters had a median survival of 10.5 dpi and a 7.6-fold greater chance of death than infected contacts (Table 2). However, ANDV was transmitted to 2 other contacts; animal c2-n2 likely evidenced a transmission chain from c2-n1, which potentially had the longest incubation time in this experiment (>18 days). Hamster c2-n2 had abundant and disseminated ANDV RNA in tissue samples (Figure 2, middle column) but was the only infected contact that failed to mount an anti-N antibody response (Figure 2, right column). Animal c6-n2 was likely infected after a late transmission from the persistent shedding of animal c6-i2. Infection of c6-n2 was strongly suggested by the increasing ANDV RNA in rectal mucosa at 40 dpi, which was above intracage cross-contamination levels (Figure 2, panel F, left column). In addition, we detected intermediate loads of ANDV RNA in 4 tissue samples, and this hamster seroconverted (Table 1; Figure 2, panel F, middle and right columns).

**Table 2:** Hazard risks of survival and relative risk of infection of the naïve cohort compared to the inoculated cohort in study of human-to-human transmission of Andes virus modeled in Syrian hamsters*

Cohort	Deaths, %	Infected, %	HR (95% CI)	p value	RR (95% CI)	p value
Naive	27	45	0.13 (0.03−0.5)	0.003	0.45 (0.2–0.9)	0.017

*HR, hazard risk; RR, relative risk.

Overall, virus replication and dissemination were not different between inoculated animals and infected contacts. Aside from hamster c6-n2, which was likely in an early phase of infection, all infected animals showed virus distribution consistent with previous studies (*7*,*11*); lung and liver samples harbored the highest ANDV RNA loads at euthanasia (Figure 1, panel F). Pairwise tissue comparison among cohorts revealed no significant differences in ANDV RNA loads in animals that became infected (Appendix Figure 3). Likewise, after adjusting incubation days in the naive cohort (Appendix Figure 4), shedding loads did not differ between infected animals of either cohort (Figure 1, panels B–C) or by route of shedding (Figure 1, panel D) or shedding duration (Appendix Figure 5). Yet, shedding onset was delayed in contact animals (by 9–30 adjusted incubation days). Other parameters (i.e., periodic weight loss and temperature variation) did not differ significantly between cohorts of infected animals (Appendix Figures 6, 7).

## Conclusions

Reports of human-to-human ANDV transmission in recent decades (*3*–*5*), which could be driven by specific mutations of ANDV (*12*), highlight the importance of this phenomenon. In a 2018–2019 outbreak in Chubut Province, Argentina, for example, 33 persons were estimated to be infected after chains of transmissions started from 1 infectious person (*5*). The potential for human-to-human transmission has drastic implications for public health. Not only is spillover from reservoirs a consideration, but human transmission chains add further complexity in outbreak settings, requiring additional control measures, potential quarantine of infected persons and contacts, and additional precautions in dealing with HCPS patients. 

We established an efficient model of ANDV transmission between hamsters and a method for monitoring infection. Moreover, because ANDV shedding loads did not differ by route in infected hamsters and oral shedding began 1 day earlier than with other routes, this model can be further simplified. We describe ANDV shedding kinetics in infected Syrian hamsters, but because of the known difficulties in Andes virus isolation (*13*), we cannot accurately determine infectious shedding kinetics. Still, infectious particles were shed from >4 of the inoculated animals, 1 of which was a persistent shedder. New World hantaviruses are thought to persistently infect and be intermittently shed from reservoir hosts (*2*). However, persistent shedding has not been reported in humans or animal models. Although ANDV RNA has been detected in body fluids of humans with HCPS (*14*), infectious virus has only been isolated from blood (presymptomatic) (*1*). Unfortunately, because of the study endpoint, we could not evaluate prolonged infectious shedding from an asymptomatic animal (c1-i1).

We also demonstrate that infectious ANDV was serially shed from an infected hamster to a naive hamster, then on to another naive hamster (cage 2). Viral shedding onset was delayed in the naive cohort, particularly for serial transmission. This delay could be because of our rudimentary adjusted incubation day threshold or could be a consequence of the transmission route, the transmitted dose (*7*), or even virus adaptation (*13*). Further studies are warranted to elucidate accurate incubation periods, transmission rates, and routes (e.g., contact, fomite, or aerosol). Increasing the experimental time frame also could have improved the results of this study. 

Disease could not be verified for 2 infected contact animals. Whether disease would have developed to an extent requiring euthanasia is uncertain. In the case of hamster c2-n2, the high viral abundance and dissemination, paired with a lack of ANDV-specific antibodies could have enabled disease progression; favorable prognosis in humans is correlated with a strong IgG response early in disease (*15*). 

In summary, our results demonstrate that the Syrian hamster is an appropriate model for horizontal transmission of ANDV. We demonstrated clear pathogenesis and further horizontal transmission in contact animals.

Appendix Additional information about human-to-human transmission of Andes virus modeled in Syrian hamsters.
