# Supplementary material for: Human-to-Human Transmission of Andes Virus Modeled in Syrian Hamsters
Source: Emerg Infect Dis. 2023 Oct;29(10):2159–63. doi: 10.3201/eid2910.230544 (PMC10521624; doi:10.3201/eid2910.230544)
Supplement: Appendix — Additional information about human-to-human transmission of Andes virus modeled in Syrian hamsters. [file 23-0544-Techapp-s1.pdf]

Article DOI: https://doi.org/10.3201/eid2910.230544

EID cannot ensure accessibility for supplementary materials supplied by authors. Readers who have difficulty accessing supplementary content should contact the authors for assistance.

Human-to-Human Transmission of Andes Virus Modeled in Syrian Hamster

Appendix

Materials and Methods

Study design

To account for unknown ANDV shedding and transmission rates between hamsters, twelve pairs of inoculated-naïve animals were used. To maximize exposure, two hamsters inoculated with ANDV were enclosed in a cage in direct contact with two naïve animals, in a total of 6 cages, as shown in Appendix Figure 1.

Animals and husbandry

Twenty-four Syrian hamsters were purchase from Janvier labs, France, and transported to the BSL-4 facility. Four hamsters of ≈3-weeks old (<100 g) were allocated into each of six IVC GR900 cages (900 cm^2, Tecniplast Sealsafe). Low-dust bedding, a shelter and nesting material were provided. Animals were acclimated for 12 days and kept in a reversed 12-hour daylight cycle, at 21°C (±1°C) and 50% (±10%) of relative humidity. Water and food were offered *ad libitum* throughout the experiment.

In vivo infection

ANDV Chile-9717869 was quantified by infecting monolayers of Vero E6 cells in 96-well plates with four replicates of six ten-fold dilutions ($1x10^{-0}$ – $1x10^{-6}$). Supernantants were harvested either at 1 hour, or days 1, 2 and 4 post-inoculation. The lowest dilution that demonstrated an increase in ANDV-RNA copies over time was estimated as an equivalent of 1 plaque forming unit (PFU-eq).

Under isoflurane anesthesia, hamsters were weighted, ear-tagged and implanted with a temperature-logging transponder (IPTT-300, Plexx). Two animals from each cage, randomly assigned to the naïve cohort (n = 12), were placed into six fresh cages. Animals from the "inoculated cohort" were inoculated with 200 PFU-eq of ANDV in 100 uL of sterile PBS via intranasal route (i.n.) and returned to their respective cages (Appendix Figure 1). One day post-inoculation (dpi), each pair of inoculated animals was placed into a fresh cage with their corresponding pair of naïve animals. All animals were observed daily to detect development of signs of disease and endpoint scoring. Animals that reached the endpoint scoring criteria were euthanized with an overdose of isoflurane and exsanguination by cardiac puncture. Surviving animals were euthanized on dpi 40. Oral and rectal mucosa was sampled every other day under light isoflurane sedation and urine was collected opportunistically. Animals were weighed every third day and the animals were moved to clean cages once per week or when the last surviving inoculated animal was euthanized.

Real-Time Quantitative PCR (RT-qPCR)

Swabs used to collect mucosal or urine samples were placed directly into 560 uL of AVL and inactivated in 700 µL of ethanol 100%. Viral RNA was extracted using a QIAmp Viral RNA Mini Kit (Qiagen) following the manufacturer's instructions. Tissue samples were individually weighed, then homogenized in RLT buffer using a stainless-steel bead and a Tissuelyser II (Qiagen) homogenizer, for 10 min at 30 Hz. Samples were centrifuged for 10 minutes at 6000 xg and the supernatant was inactivated in 600 µl of ethanol 70% per 30 mg of tissue. ANDV RNA was quantitated by qRT-PCR. Briefly, AgPath-ID One-Step RT-PCR mix (4387391, Thermo Fisher) was added to 5 µL of extracted RNA sample (elute) and analyzed in an Applied Biosystems 7500 platform. Primers (ANDVf: aaggcagtggaggtggac, ANDVr: ccctgttggatcaactggtt) and probe (ANDVp: FAM-acgggcagctgtgtctacattgga-BBQ) targeting a 162 bp fragment within ANDV-S segment (130 - 291 bp from NCBI ref. seq.: NC_003466.1) were added to 20 µL of master mix for total volume of 25 µL. Samples were incubated for 15 min at 45°C, 10 min at 95°C followed by 45 cycles of 15 sec at 95°C and 60 sec at 60°C. Samples that had cycle threshold (Ct) values ≤40 were regarded as positive. A standard curve of ANDV in vitro transcripts (10 to 10^7 copies) was included to quantify Ct values, which was calculated using the supplied software (ABI-7500 v2.3). To estimate ANDV copy numbers/g of tissue, the resulting ANDV-S copies per µL of elute used in the PCR mix, obtained from a total extraction

of 50 μL divided by the initial tissue weight, were extrapolated to 1 g. ANDV-S copies of swab samples and urine were calculated as undiluted samples. Analyses shown in Figures 1, 2, and Appendix Figure 2 were performed using ANDV-S copies detected per swab or collected urine sample, transformed to log base 10. Analyses show log10 transformation of the *y-axis* to improve visualization.

Serology

Sera collected at euthanasia were tested for the presence of anti-N-specific antibodies (Ab), as done previously (*22*). Briefly, 96-well Nunc MaxiSorp flat-bottom plates (Thermo Fisher Scientific Inc.) were coated overnight at 4°C with 100 ng/well of SNV-N protein, kindly provided by Dr. Tony Schountz (Colorado State University), in PBS. The plates were washed with PBS-T (0.1% Tween20 in PBS) for times and then blocked at room temperature (RT) for 1 hour with blocking buffer (2% BSA in PBS-T). Serum samples were 2-fold serially diluted in PBS, starting at a 1:50 dilution, and incubated for 1 hour at room temperature. After 4 washes, samples were incubated with a goat anti-hamster IgG (H+L) Ab (ab6892, abcam) at a 1:1000 dilution in PBS for 1 hour at room temperature. Plates were washed again and then incubated for 15 min with 2,2′-azino-di(3-ethylbenzthiazoline-6-sulfonate) (ABTS) two-component peroxidase substrate kit (SeraCare Life Sciences) following the manufacturer's instructions. Plates were spectrophotometrically measured at 405 nm on a Tecan Sunrise Absorbance reader (TECAN Instruments). Positive samples were determined by an optical density >3 SDs above the same dilution of ANDV-negative control sera (n = 7).

Statistical analyses

For statistical analyses, the naïve animal 1 from cage 1 (-n1) was excluded from all analyses, as it had to be euthanized due to the development of a gastric volvulus; disease unrelated to this infection model. The shedding dataset was curated by removing intra-cage cross-contamination, from inoculated to naïve animals, after determination of true animal infection (i.e., disease onset, tissue infection, shedding and seroconversion). ANDV-RNA cross-contamination due to environmental RNA or consumption of highly contaminated feces was observed in all cages and in the mucosa of all naïve animals. To compare shedding titers between cohorts in time, an adjusted incubation days (AID) dataset was generated. This excludes dpi 0 to 10 from all naïve animals except c2-n2, which was infected after a likely secondary transmission, therefore dpi 0 – 22 were excluded (Appendix Figure 4). All statistical analyses were done using

R. Descriptive statistics, such as mean (M), standard deviation (SD), median (Mdn) and sample size (N) are reported. After determination of non-normal distributions using qqplot visualization and a Shapiro-Wilk test (with and without data transformation), the non-parametric Wilcoxon signed-rank test (W) and the Kruskal-Wallis H-statistic was used to compare sample distributions of two or more independent sample groups. Kruskal-Wallis chi-squared (χ^2) and degrees of freedom (df) are reported. Pairwise comparisons and Dunn tests were used as post-hoc analysis using Bonferroni for multiple comparison adjustment and reported as adjusted *p* value. Survival analysis was calculated using the Mantel-Cox test. Hazard risk (HR) and relative risks of infection (RR) are reported. Visualizations were done using the ggplot2 package and in R curated and Adobe Photoshop CS6.

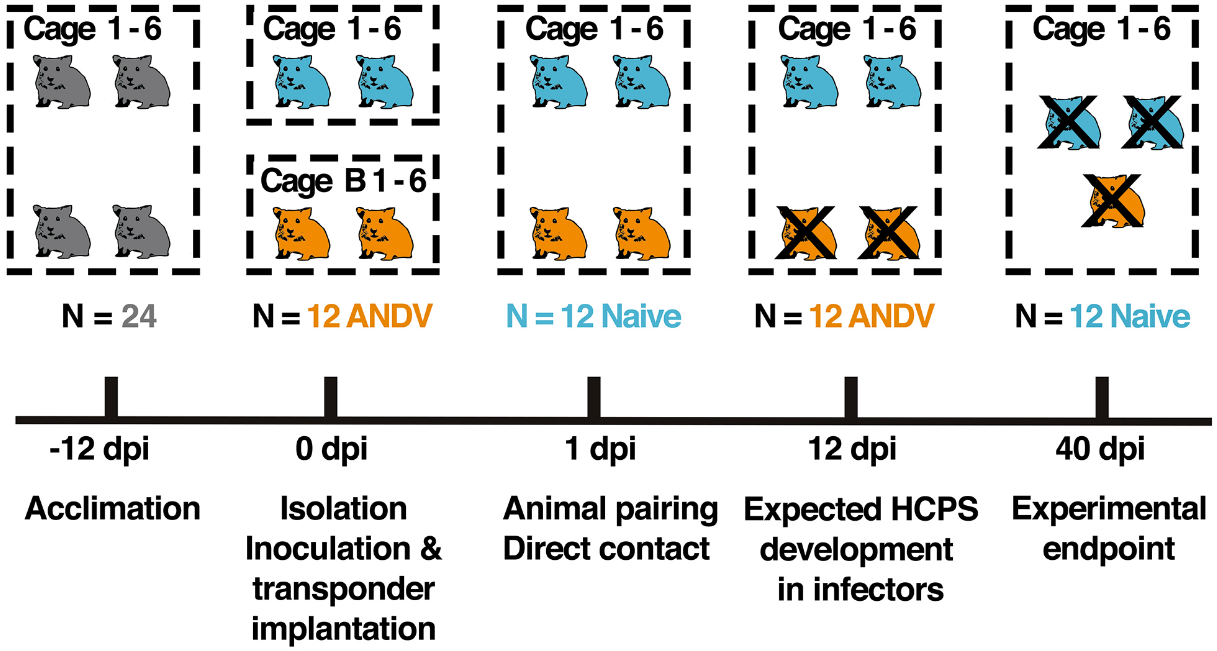

Appendix Figure 1. Study design. Inoculated hamsters (i.e., inoculated) are shown in orange and naïve animals (i.e., contacts) are shown in cyan. Animals were separated on the day of anesthesia and infection and later reunited in fresh cages at 1 dpi. Animals were expected to develop HCPS around dpi 12. All animals were observed daily for signs of HCPS and euthanasia criteria scoring. Animals that did not reach euthanasia criteria scoring were euthanized on dpi 40. Sampling to evaluate shedding was performed every other day, and weighing every third day. Animals were moved to clean cages weekly.

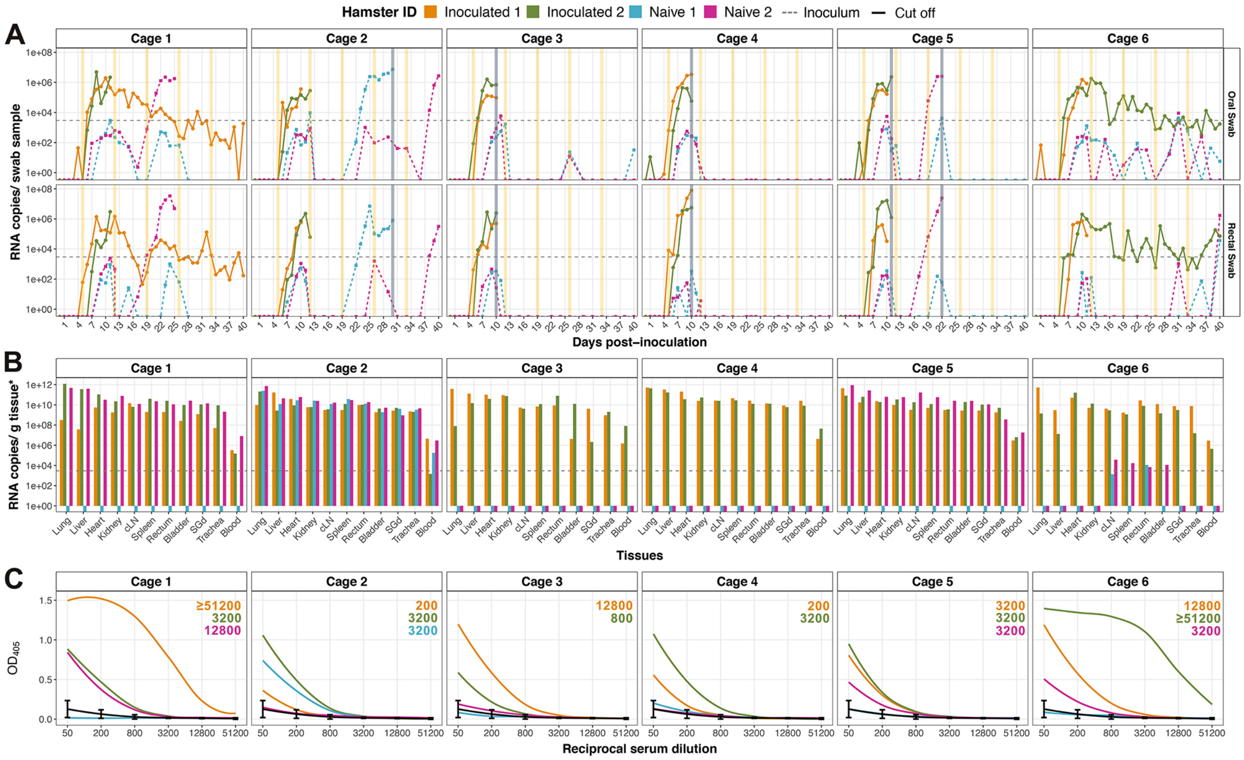

Appendix Figure 2. Timeline of Andes virus (ANDV) shedding and transmission between inoculated and naive Syrian hamster pairs from each cage. A) Shedding of ANDV RNA loads per rectal mucosa swab sample. Shedding loads of individual animals are shown as color-coded lines. Vertical shades show routine (yellow) or extra (grey) cage changes. B) Tissue distribution of ANDV RNA shown per gram of tissue or mL of blood. The dashed horizontal grey line shows the inoculum dose. C) SNV-N ELISA from serum collected at euthanasia. Anti-N serum titers are noted for animals that seroconverted. The assay cutoff is shown as a black curve with vertical line-ranges (mean +/- 3 SD) of each serum dilution. To improve figure visualization, the *y*-axis was log10-transformed (A-C). Bla, bladder; Bld, blood; cLN, cervical lymph node; Hrt, heart; Liv, liver; Lun, lung; Rec, rectum; RSD, reciprocal serum Spl, spleen.

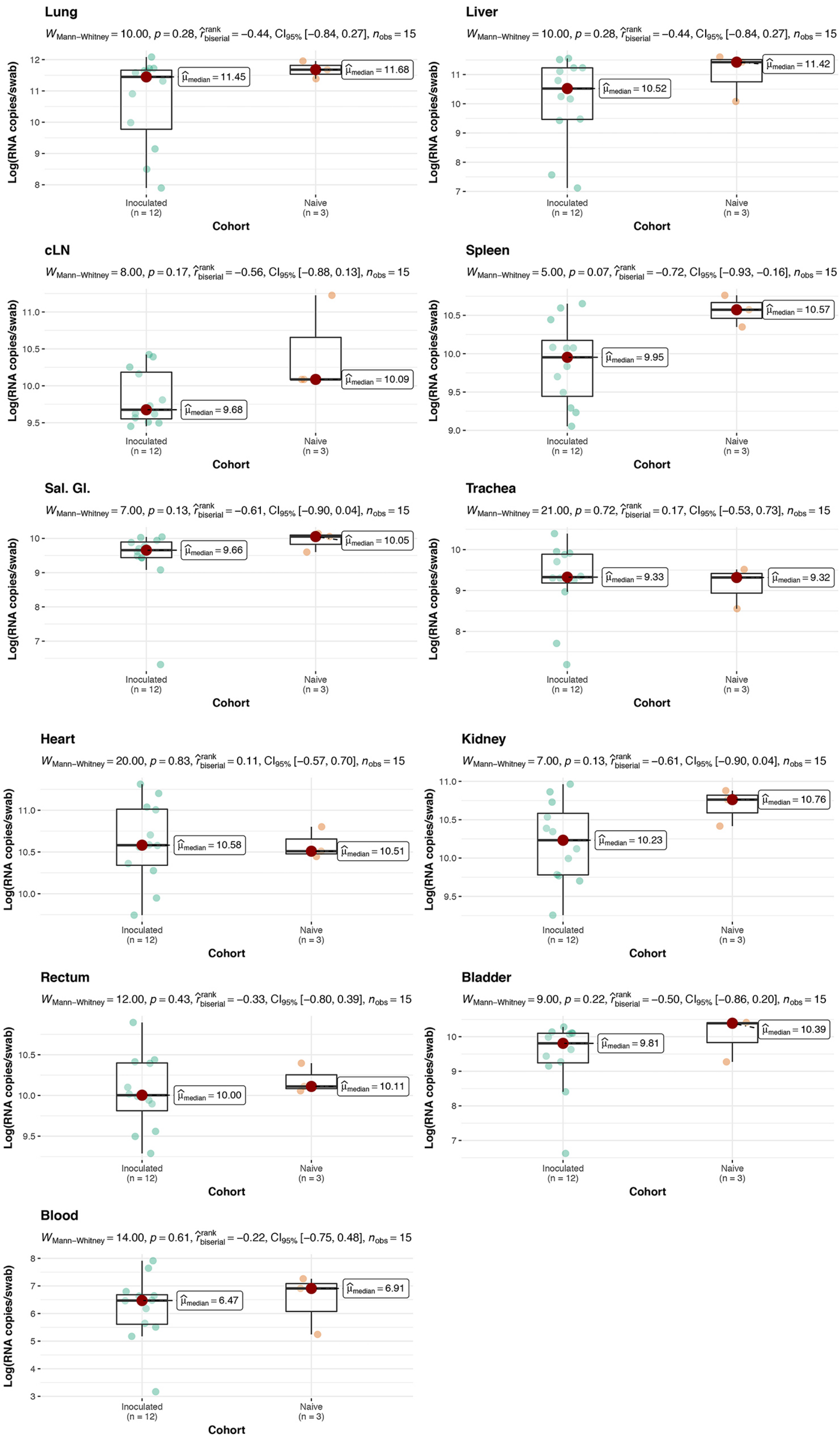

Appendix Figure 3. Pairwise comparison of ANDV-RNA loads between tissues of infected Syrian hamsters that developed HCPS. Results of descriptive statistics and non-parametric comparisons are included. No significant differences between cohorts were detected.

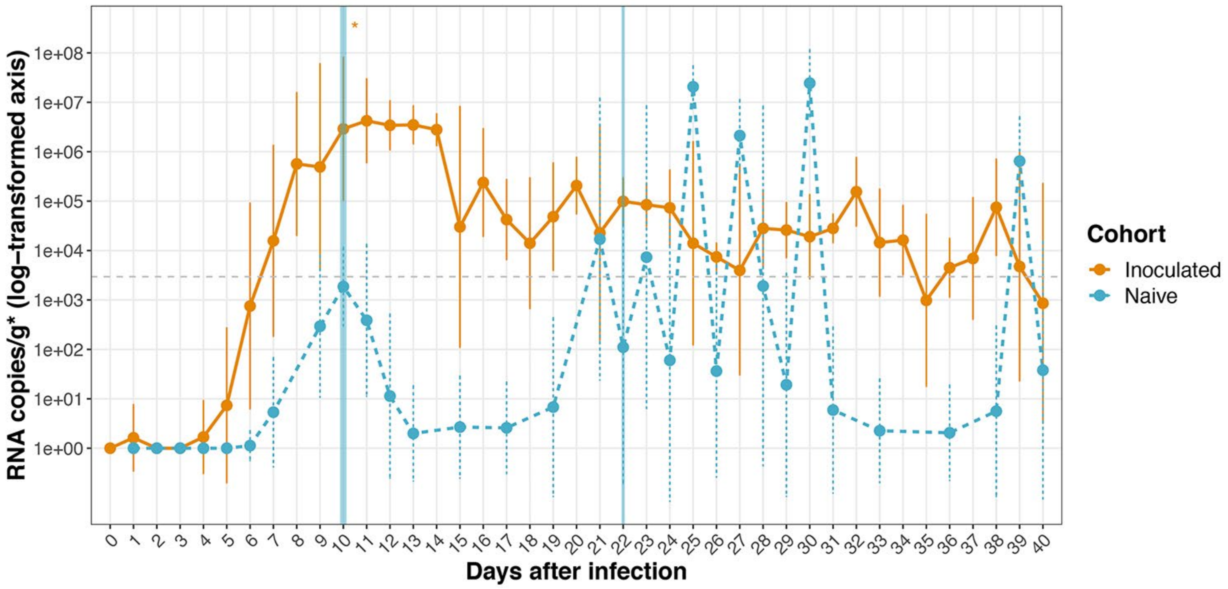

Appendix Figure 4. Adjustment of incubation days (AID) of the naïve cohort. ANDV-RNA loads, shed via all routes, are shown throughout the experimental time frame. Circles and line range show measured RNA-load average and SD for each day, respectively. Exclusion of days outside the estimated incubation are shown as vertical blue shades. Dpi 0 to 10 were excluded from all except for one naïve animal. Dpi 0 to 22 was excluded from the animal likely infected after a secondary transmission event (c2-n2). Days for exclusion were selected according to peak shedding and median survival of the inoculated cohort.

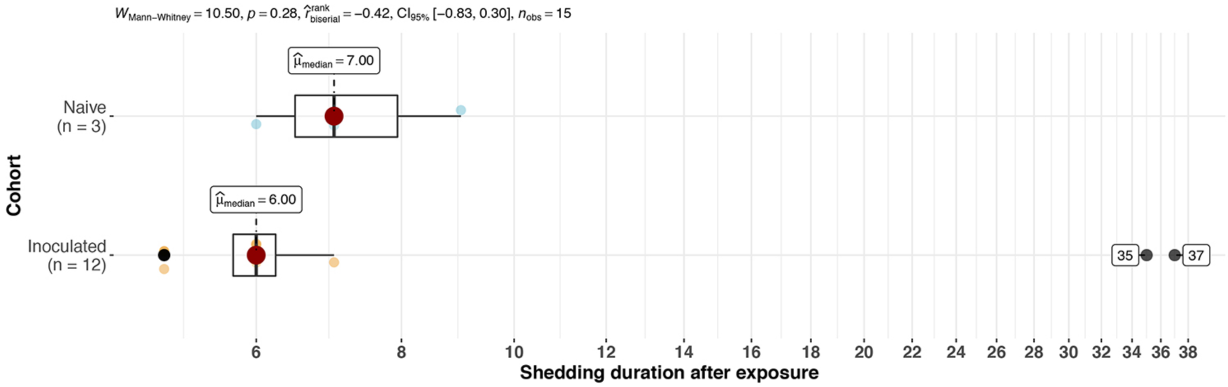

Appendix Figure 5. Comparison of shedding duration between cohorts, after adjustment of incubation days (AID). All infected animals shed virus until euthanasia. The duration of shedding was counted as the first day of shedding via any route through the day of euthanasia. Shedding duration ranged between 5–37 days (M = 10.8, SD = 11.7, Mdn = 6, N = 12) in the inoculated cohort and 6 - 9 days (M = 7.3, SD = 1.5, Mdn = 7, N = 3) in the naïve cohort (Figure S4), which did not differ significantly (W = 10.5, p = 0.284, N = 15).

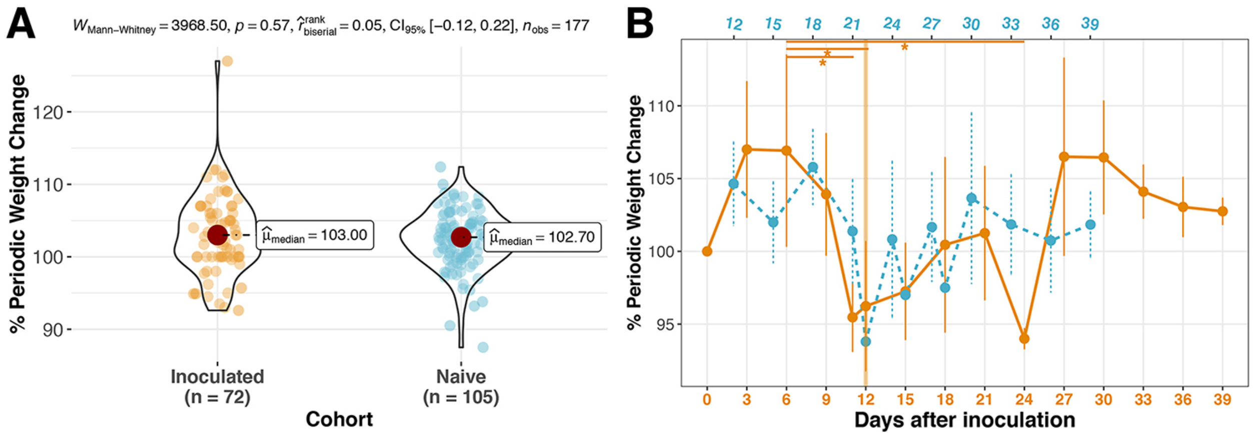

Appendix Figure 6. Percentage of periodic weight change of cohorts with AID. A) Between cohorts and B) Shown in time with superimposed AID days of the naïve cohort; The x-axis at the bottom shows the experimental schedule of the inoculated cohort and the x-axis on top show the superimposed dpi that likely reflects the incubation period for the naïve cohort. Circles and line range show average weight of a day and SD, except on dpi 10, 13, 16 of the naïve cohort, where only one animal was measured (day of euthanasia). Survival of the 2 inoculated-hamsters is shown from the vertical orange shade forward. We detected no significant PPWC differences between cohorts. However, there were differences between AID days (KW χ^2 = 45, df = 17, p = 0.0002), specifically between AID 6 with 11, 12 and 24 (p = 0.03, 0.03, 0.01 respectively). Significant differences between days (N > 2) are reported as * ≤0.05.

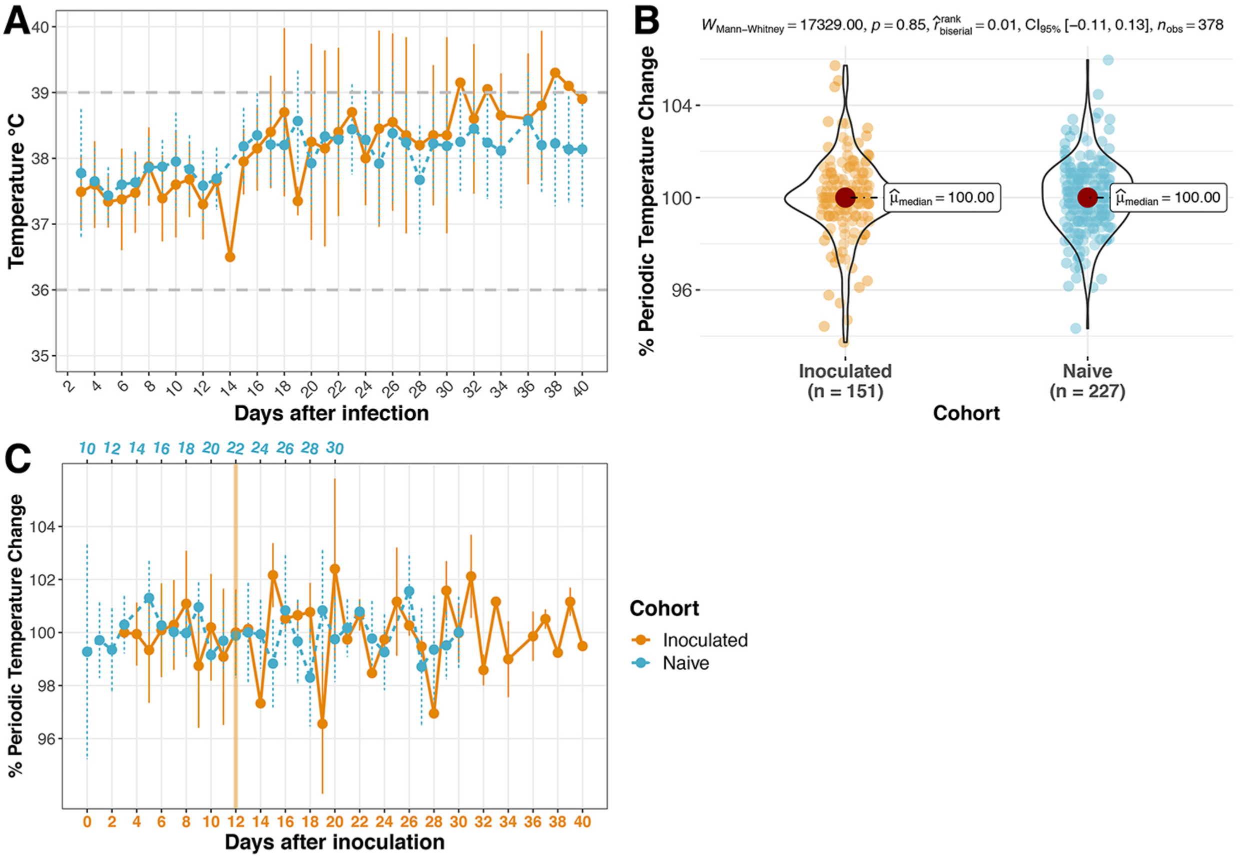

Appendix Figure 7. Daily temperature change of experimental cohorts. A) Average daily temperature (°C) shown throughout the experimental time frame. Grey dashed lines show the limits of body temperature reference values for Syrian hamsters. B) Daily percentage change comparison between cohorts with adjusted incubation days (AID). C) Daily percentage change comparison in time with superimposed AID days of the naïve cohort; the *x-axis* at the bottom shows the experimental schedule of the inoculated cohort and the *x-axis* on top show the superimposed dpi that likely reflects the incubation period for the naïve cohort. Circles and line range show daily average and SD, respectively. Survival of the 2 inoculated-hamsters is shown from the vertical orange shade forward. Periodic percent temperature change (PPTC) did not significantly differ between cohorts. PPTC variation differ between AID days (KW $\chi^2 = 53$, df = 36, p = 0.03, Figure S8C), but this was not resolved by post-hoc analyses.
